# Experimental manipulation of avian social structure reveals segregation is carried over across contexts

**DOI:** 10.1098/rspb.2014.2350

**Published:** 2015-03-07

**Authors:** Josh A. Firth, Ben C. Sheldon

**Affiliations:** Department of Zoology, Edward Grey Institute, University of Oxford, Oxford OX1 3PS, UK

**Keywords:** social network, assortativity, associations, Paridae, resilience, social interactions

## Abstract

Our current understanding of animal social networks is largely based on observations or experiments that do not directly manipulate associations between individuals. Consequently, evidence relating to the causal processes underlying such networks is limited. By imposing specified rules controlling individual access to feeding stations, we directly manipulated the foraging social network of a wild bird community, thus demonstrating how external factors can shape social structure. We show that experimentally imposed constraints were carried over into patterns of association at unrestricted, ephemeral food patches, as well as at nesting sites during breeding territory prospecting. Hence, different social contexts can be causally linked, and constraints at one level may have consequences that extend into other aspects of sociality. Finally, the imposed assortment was lost following the cessation of the experimental manipulation, indicating the potential for previously perturbed social networks of wild animals to recover from segregation driven by external constraints.

## 1. Introduction

Network approaches have long been acknowledged as an effective tool for studying animal sociality [1], and social interaction patterns recognized as imperative for understanding how selection acts upon individuals [2–4]. However, only recently have the necessary advances been made to allow large-scale tracking of individual associations and subsequent quantification of social structure [5,6]. Application of these approaches has discovered non-random social associations in numerous systems, and shown that these may be driven by a range of factors, including demographic classes [7], size [8], relatedness [9] or behavioural types [10,11]. Furthermore, social structure can show stability through time [12] and display consistency across different contexts [13–15].

Observed network structures also appear to be related to emergent processes, such as sexual selection [16] and inbreeding avoidance [17], and the spread of information [18,19] and disease [20–22]. Therefore, it is important to determine the drivers of network structure, as well as to characterize the flexibility of social networks and the relationships with population-level processes. Furthermore, the response of social networks to external perturbations or imposed constraints remains of much importance to advancing understanding of social systems and potentially animal conservation [23–25]. However, the difficulty of experimentally manipulating social associations has limited our understanding of the causes, and thus arguably the consequences, of network structure. Indeed, the lack of experiments in this area may somewhat reduce our confidence in the findings of network studies generally [26].

Fortunately, the monitoring of wild animals whose social structure is perturbed by single, incidental events has demonstrated that such factors may contribute to social segregation arising, yet this imposed assortment can dissipate as the effect of the perturbation is reduced [25,27,28]. Nevertheless, it remains unclear if the eventual loss of segregation is due to network resilience, or simply restructuring. Further, owing to the uncontrolled nature of such rare, natural experiments, it is uncertain whether such environmental perturbations cause separation or exaggerate an underlying assortment, and whether perturbed social networks change permanently or tend to return to their earlier state.

Controlled experimental approaches aimed at directly manipulating social structure have so far been limited to removal and addition of individuals. For instance, manipulations using captive fish have found social ties can be maintained despite experimentally reduced cooperation via removal [29], and that the strength of a network's links influence whether or not it is disrupted by the introduction of novel individuals [30]. Similarly, social connectivity within wasp colonies (*Ropalidia marginata*) appears resilient to substantial loss of individuals, owing to the initial redundancy within the network [31]. Yet Flack *et al.* [32] demonstrated that temporary removal of key individuals can cause multiple changes to social structure in captive pigtailed macaque (*Macaca nemestrina*) societies.

Nevertheless, in addition to logistical and ethical challenges [23], simple addition or subtraction of individuals inevitably changes both population density and composition, which may have numerous effects independent of changes in social structure [31,32]. Further, networks considering the same individuals but different kinds of interactions or associations show correlations in overall structure and dyadic relationships [13–15,33], yet removal of individuals does not allow investigation of this. However, these cross-context correlations may have practical implications for animal welfare and conservation, particularly in predicting how disturbance to one social dimension may have implications for another [33], as well as being of interest for our understanding of sociality and general network theory [15].

In this study, we use a novel experimental method to enable fine-scale manipulation, and subsequent assessment, of a wild passerine bird social system. By controlling which birds were allowed to forage together at different electronically controlled feeding stations, we imposed social assortment and segregation between spatially affiliated and previously associated individuals. Assessing the social associations at these sites before, during and after manipulation enabled us to explicitly determine the flexibility and recovery of the social structure subject to external constraints. Finally, through independently monitoring associations at unrestricted ephemeral food patches, as well as at nest-boxes as individuals prospected for breeding territories, we determine whether a causal relationship exists between these different contexts, and how influences at one level may have carry-over effects into other components of sociality.

## 2. Material and methods

### (a) Study system

This work took place as part of larger study examining the social ecology of wild birds in Wytham Woods, Oxford, UK (51°46′ N, 1°20′ W) [e.g. 11]. Great tits (*Parus major*), blue tits (*Cyanistes caeruleus*), marsh tits (*Poecile palustris*), coal tits (*Periparus ater*) and Eurasian nuthatches (*Sitta europaea*) were caught either at nest-boxes during the breeding season, or by mist-netting through the winter to include immigrants as well. Upon capture, birds were fitted with a standard metal British Trust for Ornithology (BTO) ring as well as a unique passive integrated transponder (PIT) contained in a plastic leg ring. As part of the core work, sunflower seed feeding stations equipped with radio-frequency identification (RFID) antennae (Dorset ID, Aalten, The Netherlands) were deployed in a stratified grid at 65 fixed locations throughout Wytham Woods; these were opened from pre-dawn until after dusk each weekend from September to February, and allowed detection of the time and location of individual's feeding attempts upon reading their PIT tag [11].

### (b) Selective feeder sites

**Figure 1.**
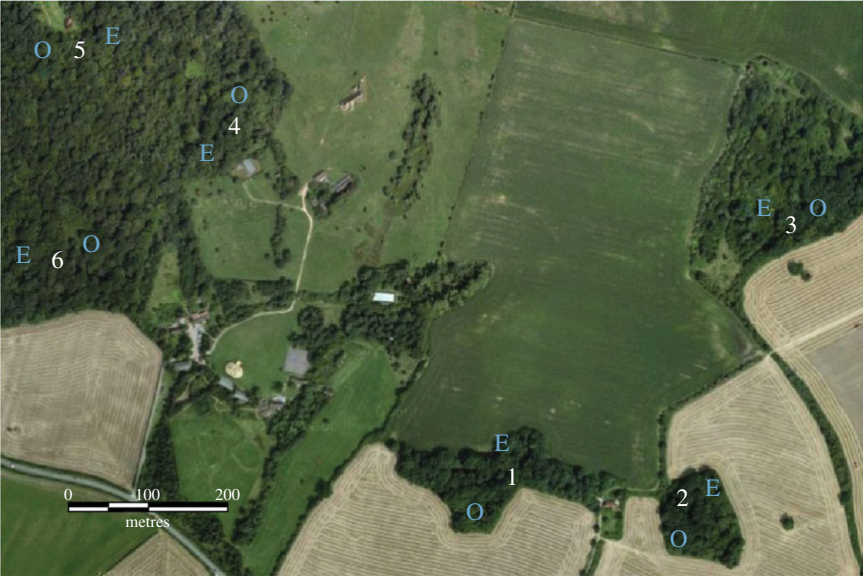
Aerial photo of study area, marked to indicate key features. Numbers indicate original selective feeder site locations. ‘E’ shows where feeders which fed only even tagged individuals were positioned during the experimental manipulation, and ‘O’ shows where feeders only allowing odd tagged individuals were located.

The current experiment was conducted between November 2013 and April 2014 in neighbouring areas of broadleaf deciduous woodland on the southern edge of the main study site (figure 1), partially separated from it by an area of scrub and arable land. These form part of the long-term study system described above and contain six of the 65 feeder stations. In November 2013, the six standard feeders were replaced with experimental feeder stations (‘selective feeders'). Selective feeders followed the same design as the standard feeders but had a clear flap locked in position over the feeding hole. The unlocking mechanism, which would allow a bird access, was controlled by a programmable circuit board (Stickman Technologies, Southampton, UK) and was only activated when the antenna registered a PIT tag number specified to be granted access (specified prior to the experiment—see the electronic supplementary material). By this means, rules controlling which individuals had access to a feeding station could be imposed automatically. During the course of this study, these automated feeders were open every day, from pre-dawn until after dusk. Initially, all tagged individuals were granted access to all the feeders; this period is referred to as ‘pre-manipulation’. After 40 days' acclimation, the experimental treatment was applied by introducing a selective regime, controlling individual access to feeders. Two selective feeders replaced each of the initial feeders, both placed approximately 50 m from the initial location (figure 1). In each case, the antenna on the feeder recorded all visits to the feeder, but one feeder within each pair was programmed to grant access only to individuals with a PIT tag ending in an odd digit, while the other would only allow access to even-digit PIT tags. This treatment enabled us to split the population into two randomly selected, equally sized groups. This time period is referred to as ‘during manipulation’. After 90 days' exposure to this condition, these 12 feeders were reset to allow access to all tagged individuals, which are referred to as the ‘post-manipulation’ period. This final period was limited to 6 days in order to minimize the impact of supplementary feeding on breeding performance and behaviour.

### (c) Ephemeral food patches

We aimed to test whether the constraints imposed at the selective feeder sites were reflected in the association patterns at unrestricted, ephemeral food patches. These were created by putting four small sunflower seed feeders [18], equipped with RFID-reading antennae (Francis Instruments Ltd., Cambridge) in randomly selected positions throughout the study area (with the constraint that they were at least 50 m from a selective feeder site). These collected data on all birds visiting and were removed after 4 days. This was carried out once during the pre-experimental period and four times during the experimental treatment, with a 10-day interval between trials. It was not repeated in the post-manipulation period as we aimed to limit interference during this shorter time period.

### (d) Nest-box recording

We also examined whether the manipulation of individual access to feeding sites also influenced which individuals would be likely to encounter one another during breeding site prospecting. We fitted nest-box RFID recorders (Dorset ID, Aalten, The Netherlands) to a randomly selected one-third (44/133) of the nest-boxes located in the largest continuous area of woodland (figure 1). These scanned for PIT tags three times per second and recorded any individuals that landed on the entrance hole while prospecting for breeding territories. This began before any nesting attempts were recorded, 12 days before the removal of the selective feeder sites (in March 2014), and continued for 24 days in total to April 2014. We did not monitor nest-boxes in the pre-manipulation period as territory prospecting only begins in late winter/early spring.

### (e) Social network construction

PIT tag detections from both types of feeder provide a spatio-temporal data stream, which shows intermittent bursts of activity as flocks of individuals come in to feed. Foraging flocks (groups) were detected using a Gaussian mixture model. This model exploits the non-homogeneous profile to statistically assign each visit to the group for which it has the highest probability of belonging to [34]. We also aimed to infer associations taking place while individuals prospected for breeding territories. Typically, only a small number of individuals visit each nest-box each day (mean = 1.7), and preliminary analysis of a larger dataset (B. Verhelst & B. C. Sheldon 2012, unpublished data) found individuals land on the entrance numerous times, usually spanning over 4 h on average, with this peaking between 8.00 and 12.00. Also, birds landing on the entrance hole of a nest-box typically engaged in activity in the surrounding vegetation (J. A. Firth 2014, personal observation). Thus, groups were defined simply as individuals detected on the same nest-box during the same day, and co-memberships represent individuals that overlapped in nest-box exploration patterns during the same day. Although this is a longer temporal period than for groups defined from foraging flocks, the spatial scale (44 boxes within one area of woodland) is of much higher resolution. Networks were calculated from these group-by-individual matrices using the half-weight index [35]. While our approach essentially relies on the gambit of the group for inferring associations [36], the extensive sampling employed and use of weighted networks in further analysis reduces the limitations of this approach [37–39].

### (f) Network assortativity

We predicted that the experimental manipulation would impose assortment by tag type (i.e. birds with even pit tag numbers would be likely to be associated with other individuals with even tags, and odd-tagged individuals with other odd-tagged individuals) within the network. The degree of such homophily within the network was measured using the widely used assortativity coefficient [40,41] that has recently been extended to incorporate weighted associations using the R package *assortnet* [42,43]. Positive values demonstrate assortativity, with perfectly segregated networks scoring 1, and negative values represent disassortment. Perfectly integrated networks do not always score −1 as the minimum value depends on the number of node types and relative number of ties within each group [41]. The associated standard error was calculated using jackknifing [41,43]. However, to account for the non-independence of network data, the assortment coefficients were compared with results calculated from 10 000 randomized networks generated via node permutation (i.e. maintaining the node labels while shuffling the rows and columns) of the considered network, which allows permutation of individuals' tag types while maintaining the observed association patterns, levels of gregariousness and distribution of types [44,45].

### (g) Network consistency

As well as assessing levels of imposed assortment by tag type, we examined network consistency. First, we used Mantel tests [46,47] using the R package *ecodist* [48] to compare the pre-manipulation social network with the entire networks created both during and after the manipulation. As the post-manipulation network was derived from a smaller sampling period than the during-manipulation network and was more temporally separated from the pre-manipulation period, we constructed networks for each consecutive 6-day time window, and compared each of these with the final 6 days of the pre-manipulation network and with the 6 days of the post-manipulation network. Second, Mantel tests were used to examine the relationship between the association patterns at the selective feeder sites and those at the ephemeral food patches and nest-boxes. We compared networks derived from each 4-day deployment of ephemeral food patches with the networks at the selective feeder sites 4 days before the food patches were deployed (to avoid any interference that the food patches may have had). We also compared the nest-box network with the selective feeder networks. To do this, we used the final 6 days of the manipulation period and the 6-day post-manipulation period. We split both of these periods into two 3-day networks, and comparisons were drawn between the selective feeder site networks and the nest-box networks simultaneously in time. In all cases, Mantel tests were run including only individuals observed in both of the matrices that were being compared.

A relationship between the social associations at the selective feeder sites with either of the other two social contexts might simply be driven by the spatial distribution of individuals, where those sharing the same home range could be more likely to encounter one another over all dimensions [12]. Therefore, we employed a multiple regression quadratic assignment procedure with double semi-partialing (MRQAPDSP) [49] using the R package *asnipe* [50], which allows a single dependent matrix to be regressed against multiple independent matrices. The dependent matrix was set as the social association matrix of the context of interest, while the independent matrices were (i) a binary spatial range overlap matrix, where only dyads occurring at the same selective feeder site (figure 1) over the considered time period (i.e. 4 days for when comparing to ephemeral patches, 3 days for nest-boxes) were scored 1, and (ii) a social association matrix at the selective feeders. This allowed us to determine the relative contribution of both the fine-scale social associations at the selective feeder sites the spatial range overlap of individuals to the associations within the other contexts.

## 3. Results

Over the entire study period the selective feeder sites collected 3.06 million records of visits from 376 individuals. From this, 67 027 groups were detected using the Gaussian mixture model (see Material and methods) with a mean size of 3.8 ± 0.01 while the typical flock size (i.e. group size experienced by the average individual) was 5.8, and these gathering events lasted an average of 191 ± 0.6 s. 339 birds were detected during the experimental manipulation period; these individuals contributed 3.04 million (more than 99%) of the total records, and included all the study species and showed frequent movements around the sites (electronic supplementary material, tables S1 and S2). Since these individuals were subject to the treatment, they were included in all further analyses. However, we also ran the analysis considering (i) only the primary Wytham study species, *Parus major* (1.48 million records, 136 individuals), and (ii) only those individuals that were detected on the selective feeders before, after and during the manipulation, therefore indicating they were using the study area over the entire period (2.51 million records, 109 individuals). The results using these subsamples demonstrated similar effects as those discussed below, but differed in levels of statistical significance, probably due to the reduced sample size (electronic supplementary material, figure S2).

### (a) Experimentally imposed social assortment

**Figure 2.**
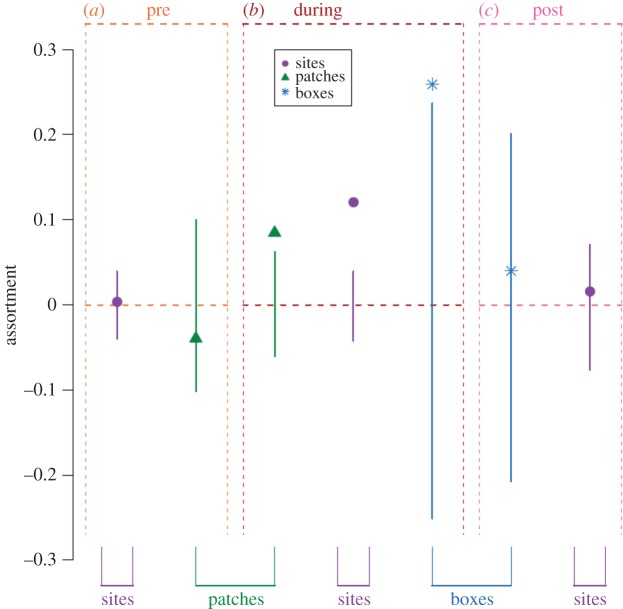
The observed level of assortment by PIT tag type in the system over the different periods and social contexts. Vertical lines show the 95% range of the assortment coefficients calculated from permuted data. Dots indicate the observed assortment coefficient. Colours of lines and point types illustrate data from different contexts (purple circle, selective feeder sites; green triangle, ephemeral patches; blue star, nest-boxes; base × axis). (*a*) ‘Pre-manipulation’ period. (*b*) ‘During-manipulation’ period. (*c*) ‘Post-manipulation’ period.

No assortment by tag type was detected at the selective feeders in the period before the experimental treatment began (weighted assortativity coefficient: *r* = 0.003 ± 0.007, *n* = 204) and this did not differ significantly from the assortment values generated from the permuted networks (figure 2*a*). A similar pattern was also found at the ephemeral food patches (*r* = −0.039 ± 0.031, *n* = 53; figure 2*a*). Therefore, the assumption that associations are random with regard to tag type prior to the experimental manipulation, and that any changes are the result of the treatment, appears valid.

During the manipulation period, relatively strong assortment by tag type was observed at the selective feeders (*r* = 0.121 ± 0.005, *n* = 339), which was also significantly higher than the assortment observed in the randomized data (figure 2*b*). Therefore, the experimental treatment successfully imposed assortativity by tag type, so that birds of the same type became more strongly associated with each other than with individuals of the opposite type.

Over the experimental manipulation period, 141 out of 147 (96%) individuals using the ephemeral patches and 54 out of 64 (84%) individuals recorded prospecting at the sampled nest-boxes were also detected at the selective feeder sites. This allowed us to assess whether the segregation imposed at the selective feeder sites was carried over into other aspects of sociality. Indeed, assortment by tag type was observed in both of these independently measured contexts (patches: *r* = 0.084 ± 0.01, *n* = 141; boxes: *r* = 0.259 ± 0.014, *n* = 54) and this was also significantly higher than in the permuted data (figure 2*b*). Therefore, we found clear evidence of carry-over of the experimentally imposed effect onto the associations occurring in non-manipulated social contexts.

Following the cessation of the manipulation, the assortment observed in the associations at the selective feeders returned to its initial low value and was once again not significantly different from zero (*r* = 0.016 ± 0.010, *n* = 124) or the randomized networks (figure 2*c*), therefore demonstrating a recovery from the imposed segregation. This was also mirrored in the associations at the nest-boxes after the manipulation period (*r* = 0.040 ± 0.021, *n* = 74; figure 2*c*).

This apparent recovery (i.e. loss of the experimental condition) was not due to a lack of power to detect assortment at the selective feeder sites over the shorter 6 days of the post-experimental period, as assortment during the experimental period was detected in all but one case when split into 6-day periods (electronic supplementary material, figure S3). Moreover, the post-experimental period had lower assortment than any of the 6-day experimental period sections (electronic supplementary material, figure S3).

### (b) Network consistency

**Figure 3.**
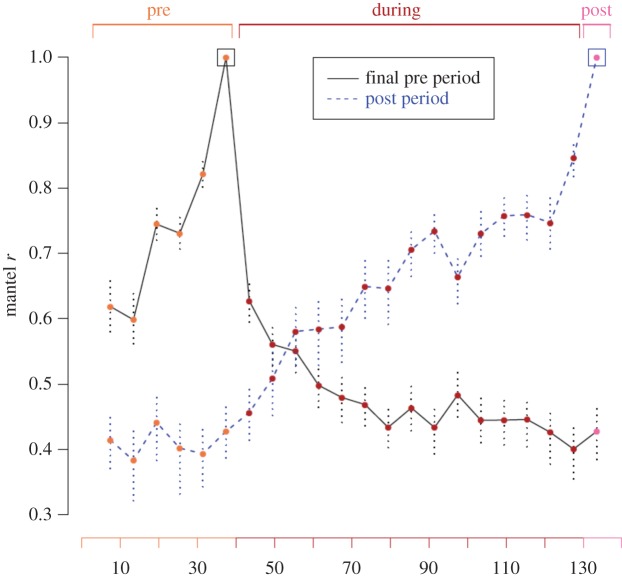
Mantel test results comparing each 6-day network to the final 6-day network of the pre-experimental period (solid black line) and to the 6-day post-experimental network (dashed blue line). Point colour denotes experimental period. Boxes show point of comparison. Vertical dotted lines indicate 95% range of Mantel test statistic.

There was no evidence that this loss of the experimentally induced assortment at the selective feeders was driven by a reversion to the same social network structure as that prior to the manipulation, as the Mantel correlation between the pre-manipulation network and the post-manipulation network was not higher than the correlation between the pre-manipulation network and the during-manipulation network (Mantel test—pre versus during: *r* = 0.55, lower = 0.45, upper = 0.67; pre versus post: *r* = 0.49, lower = 0.19, upper = 0.63). This remained true when networks were created from 6-day periods to assess consistency over more equal sampling periods and time scales. Here, the final 6 days of the pre-manipulation period did not predict the post-manipulation network more strongly than it predicted 6-day ‘during-manipulation’ networks (figure 3). Therefore, no evidence of a ‘reset’ to previous specific dyadic associations was evident following the experiment. Similarly, the post-manipulation network was not more strongly related to the 6-day pre-manipulation networks than it was to the 6-day during-manipulation networks (figure 3). Beginning the manipulation immediately and strongly affected the network, but it monotonically and gradually approached its post-treatment structure, just as the pre-treatment networks approached the final pre-treatment structure (figure 3). These patterns remained even when only considering individuals that appeared in every 6-day period (electronic supplementary material, figure S4).

Associations at the set selective feeder sites were highly consistent with those at ephemeral food patches and during nest-box prospecting (table 1). Further, consistency levels between the social network at the set selective feeder sites with the other contexts showed no large differences between when the experimental manipulation was applied and in its absence (table 1). MRQAPDSP models showed that the social association matrix at the selective feeders had a large significant effect on the social association network created from the nest-boxes (table 1). Yet a matrix simply measuring range overlaps between individuals (spatial range overlap matrix) yielded no significant relationship (table 1). Therefore, the consistency of the selective feeder site network with the nest-box network was driven primarily by fine-scale social associations, rather than simply the spatial distribution of individuals. Similarly, although spatial range overlap had a significant effect on associations at ephemeral food patches, the selective feeder social network had between 3- and 10-fold greater effect (table 1). It should be noted that the spatial location of individuals may also be related to social choices at a coarser scale, as individuals moved freely around the selective feeder sites (electronic supplementary material, table S1) and may occur in particular areas due to the other individuals that are there. Over the 6-day periods considered for social network consistency at the selective feeder sites (figure 3), spatial range overlap between individuals remained highly consistent (approx. 0.65 Mantel *R*; electronic supplementary material, figure S5), and the manipulation did not influence this (electronic supplementary material, figure S5).

**Table 1.** Mantel (column 4) and MRQAPDSP (columns 5 onwards) results of the relationship between the set selective feeder site social network and ephemeral patches and nest-boxes networks over the different periods (in relation to when the manipulation was applied). Mantel *R* gives 95% range, and test statistic in bold. MRQAPDSP results show estimate and *p*-value for (i) social networks and (ii) spatial co-occurrences, along with full model statistics.

context	period	days	Mantel *R*	social coeff.	social *p*.	spatial coeff.	spatial *p*.	full F.	full *R*^2^
patches	pre	34–37	0.24–**0.33**–0.40	0.45	<0.001	0.012	0.36	46	0.108
	during	75–78	0.35–**0.40**–0.45	0.306	<0.001	0.11	<0.001	460	0.334
		89–92	0.38–**0.46**–0.51	0.393	<0.001	0.037	<0.001	147	0.228
		103–106	0.28–**0.37**–0.43	0.367	<0.001	0.063	<0.001	149	0.195
		117–120	0.35–**0.40**–0.45	0.338	<0.001	0.031	<0.001	141	0.18
		124–126	0.36–**0.48**–0.56	0.783	<0.001	0.008	0.718	55	0.222
boxes		127–129	0.31–**0.38**–0.43	0.559	<0.001	0.021	0.338	21	0.138
	post	130–132	0.39–**0.47**–0.55	0.745	<0.001	0.002	0.863	85	0.221
		133–135	0.31–**0.41**–0.51	0.665	<0.001	−0.012	0.322	53	0.166

## 4. Discussion

Using a novel social network experiment, we demonstrated how external factors can govern associations between individuals. The effect of altering the distribution of resources available to different individuals within the same community was carried over into other aspects of social structure, yet the imposed segregation was lost following removal of the treatment. These findings were obtained by imposing rules about which individuals could forage together at different feeding sites, within a large community of wild birds fitted with RFID tags.

Monitoring wild animals subject to particular perturbations has already been extremely valuable in understanding how external factors may shape social structure, such as the observation of two distinct groups existing within a single population of bottlenose dolphins (one that fed from prawn trawlers and another that did not) [25,27]. Yet individuals may respond differently to such environmental effects based on individual differences (e.g. cognition or learning strategies), and separate groups may exist even before the perturbation. Therefore, by using a controlled experiment that determines individuals' resource access entirely randomly with respect to innate differences and prior social ties (figure 2*a*), we demonstrate that external factors alone are indeed capable of manipulating social structure. Furthermore, this imposed segregation is maintained despite continued spatial range overlap between individuals and is transferred to non-manipulated social contexts (figure 2*b*).

This flexibility provides insight into how systems with high fission–fusion dynamics, such as these birds [51], can respond to perturbations, and may differ from that, for example, of dolphin societies, where numerous strong relationships may be present [25,27]. However, even within this system, previous work has indeed revealed non-random social network structure, as well as relationships to individual traits, such as sex and personality [11]. Also, while numerous relatively weak bonds exist, such as interspecific associations [43,52], even these are known to be important (for example, to information flow [18]), and strong, non-random bonds, such as mated pairs also occur [34,51]. Further research is now needed to clarify how the strength or type of association determines their vulnerability to perturbation, the consequences of this, and how mechanisms of decision-making at the individual level relate to higher- order network structure.

We found that the experimentally driven partitioning of birds according to tag type was lost upon removing the treatment (figure 2*c*; electronic supplementary material, figure S3). We demonstrated that this recovery from the imposed segregation did not comprise a return to a social structure identical to that prior to the manipulation (figure 3), but rather simply allowed free mixing of individuals once again. This also may be expected from the system's fission–fusion dynamics, as the stability of such groups is thought to be reasonably low [51]. Hence, the social network may rapidly return to expressing non-manipulated properties following the loss of previous constraints. This is in line with the findings among bottlenose dolphins that removing the external factor resulted in the loss of segregation [25].

Finally, although the network recovered from the segregation once it was no longer imposed, association patterns during the treatment phase were maintained across different contexts. The social network at the selective feeder sites closely matched those at ephemeral, non-restricted food patches and at nesting sites during breeding territory prospecting (table 1). Although there seems little previous work in birds, research on mammals has suggested relationships between associations within different contexts may be driven by the spatial distribution of individuals [13,14,33]. Here, by considering spatially affiliated individuals, and subsequently separating the effects of spatial range overlaps and social association, we showed that the experimentally imposed associations at the selective feeder sites were a much stronger predictor of the associations within the other contexts than the simple range overlaps of individuals (table 1). Previous studies of mammals have suggested that changes or perturbations to interactions within one context may influence associations within another [33,53,54]. Yet experimental manipulations influencing associations within one context alone were needed to assess whether these can directly influence associations in another, and indicate whether perturbations or constraints within single contexts may carry over. Here, we show that the arbitrary imposed constraints on social associations at the selective feeder sites carry over to the networks in open ephemeral food patches and during territory acquisition (figure 2), thus clearly demonstrating the causal relationship between social contexts, and the potential for external factors which influence associations within one dimension to impact upon other aspects of sociality.

Along with testing the causes of social network structure, experiments directly manipulating social structure will also enable the causal links to be established between social networks and emergent processes, such as mating decisions and the transmission of disease and information [16–22]. As an example, Aplin *et al.* [18] showed that in mixed flocks of tit species (Paridae; as studied here) discovery of novel food patches increased with the centrality of an individual, and appeared to transfer between social companions. This suggests a relationship between social structure and information transfer. However, further work by Aplin *et al*. [11] has shown that the network of one of the study species (*Parus major*) is significantly assorted by standardized measures of exploration behaviour, a trait that is also known to be linked to foraging behaviour [55], and also that relatively fast explorers occupy more central network positions [11]. Therefore, rather than social structure being instrumental in determining resource discovery, it is possible that the association is founded on an underlying trait correlated with both. Thus, while correlational evidence indicates social networks may affect emergent processes [16,18–22], experiments directly manipulating associations are needed to establish how causal these links are, and at what scale they operate.

In this study, we have demonstrated that these direct fine-scale manipulations of social structure are indeed possible. This revealed that social networks are flexible, and external factors can impose social segregation between spatially affiliated individuals. Furthermore, this assortment is also manifested within social contexts where the constraint is not enforced, thus demonstrating the potential for external factors to have knock-on effects into multiple aspects of sociality. Nevertheless, recovery from the externally imposed segregation is achieved rapidly in this fission–fusion system. Future experiments directly manipulating various social networks will enable explicit assessment of other properties of social structure, as well as links to wider processes.

## Supplementary Material

Supplementary Information

## Data accessibility

Data are available on Dryad (doi:10.5061/dryad.dh777).
