# Supplementary material for: Experimental manipulation of avian social structure reveals segregation is carried over across contexts
Source: Proc Biol Sci. 2015 Mar 7;282(1802):20142350. doi: 10.1098/rspb.2014.2350 (PMC4344146; doi:10.1098/rspb.2014.2350)
Supplement: Supplementary Information [file rspb20142350supp1.docx]

**Supplementary Information**


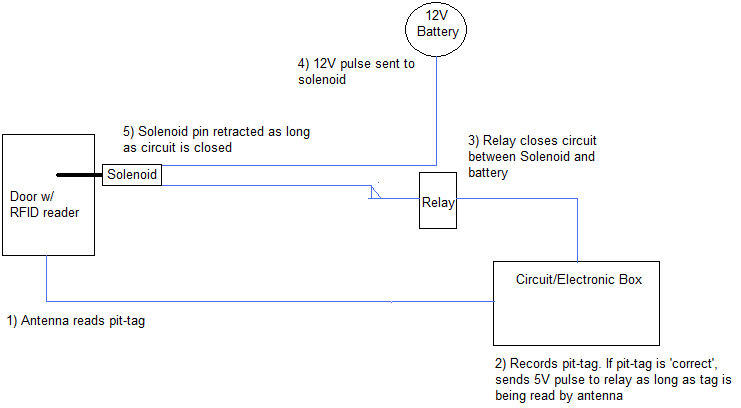


Figure SI 1. Logic flow of selective feeder. A hinged, clear, door/flap over the feeding hole is held shut by a vertical solenoid pin. Upon the antenna reading a PIT-tag, the printed circuit board (PCB) (Stickman Technologies Inc. Southampton, U.K.) determines whether this is specific tag is allowed access, and if so it activates the relay that closes the circuit between the 12V battery and the solenoid, causing the pin to be retracted. Upon the bird leaving, the door/flap closes. The relay breaks once the tag is no longer being read by the antenna and the solenoid pin is reset to its original position, holding the door shut.

Table SI 1. The number of 339 considered individuals recorded at each site (diagonal) (See Figure 2; where ‘E’ and ‘O’ at single sites are grouped together) and the number of individuals shared between each pair of sites (Off-diagonals).

|  | 1 | 2 | 3 | 4 | 5 | 6 |
| --- | --- | --- | --- | --- | --- | --- |
| 1 | 73 |  |  |  |  |  |
| 2 | 48 | 71 |  |  |  |  |
| 3 | 16 | 17 | 57 |  |  |  |
| 4 | 32 | 30 | 13 | 217 |  |  |
| 5 | 16 | 18 | 7 | 109 | 133 |  |
| 6 | 12 | 13 | 7 | 63 | 44 | 84 |

Table SI 2. The species composition of the 339 considered individuals at each site. Final column shows number of individuals detected over all sites

|  | 1 | 2 | 3 | 4 | 5 | 6 | All |
| --- | --- | --- | --- | --- | --- | --- | --- |
| Blue tit | 37 | 30 | 30 | 121 | 60 | 40 | 178 |
| Great tit | 32 | 36 | 23 | 81 | 59 | 36 | 136 |
| Marsh tit | 3 | 4 | 2 | 7 | 8 | 5 | 12 |
| Nuthatch | 1 | 1 | 2 | 4 | 2 | 0 | 7 |
| Coal tit | 0 | 0 | 0 | 4 | 4 | 3 | 6 |

Table SI 3. Sample size information for Figure 3. The total number of individuals within each of the 22 6-day periods (First row), along with the number shared with the ‘final pre experiment’ period (Second row) and the ‘post experiment’ period (Third row). Colours denote the experimental period as in Figure 3 i.e. orange = pre-experiment, red = during experiment, pink = post-experiment

| PRE | | | | | | DURING | | | | | | | | | | | | | | | POST |
| --- | --- | --- | --- | --- | --- | --- | --- | --- | --- | --- | --- | --- | --- | --- | --- | --- | --- | --- | --- | --- | --- |
| 131 | 135 | 126 | 135 | 145 | 149 | 165 | 174 | 152 | 164 | 160 | 172 | 154 | 164 | 152 | 143 | 145 | 151 | 139 | 124 | 125 | 122 |
| 103 | 109 | 113 | 116 | 124 | 149 | 130 | 126 | 117 | 118 | 118 | 120 | 112 | 112 | 106 | 106 | 103 | 106 | 99 | 93 | 93 | 92 |
| 82 | 88 | 82 | 85 | 94 | 92 | 101 | 101 | 95 | 102 | 109 | 108 | 105 | 108 | 109 | 108 | 110 | 108 | 112 | 109 | 111 | 122 |


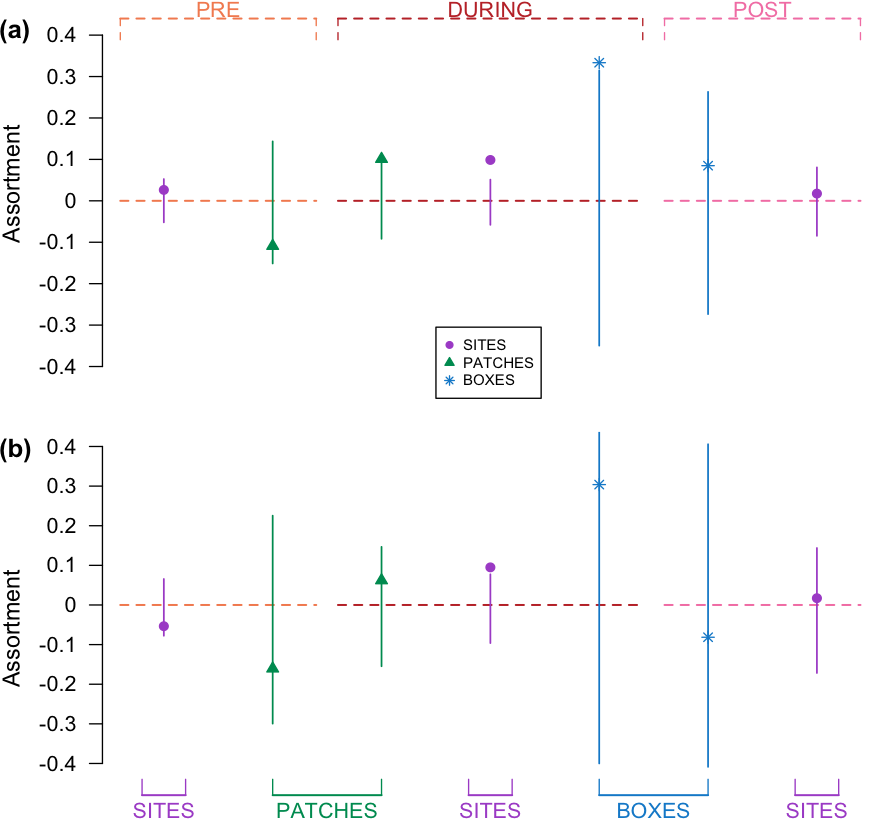


Figure SI 2. Results of supplementary analysis considering subsamples of individuals (See Figure 2 for all individuals, and Methods for description). The observed level of assortment by tag type in the system over the different periods and social contexts. Vertical lines show the 95% range of the assortment coefficients calculated from permuted data. Dots indicate the observed assortment coefficient. Colours of lines and point types illustrate data from different contexts (Purple circle = selective feeder sites, Green triangle = ephemeral patches, blue star = nest boxes; base x axis). A) Only individuals detected at the selective feeders before, during, and after the experimental manipulation. B) Only great tits (*Parus major*).


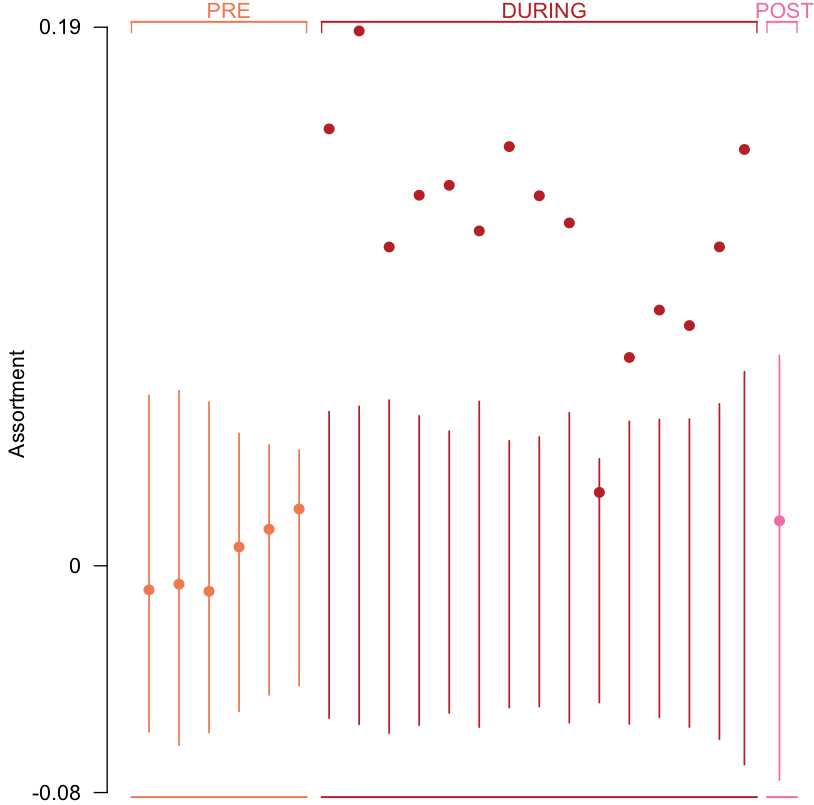


Figure SI 3. Assortment by tag type at the selective feeder sites analyzed in 6 day periods over the three measures. Vertical lines show the 95% range of the statistics derived from permuted data. Dots indicate observed value. Where the vertical lines overlap the stars there is no significant difference between the observed statistic and the null models. Colours illustrate different periods (x axis).


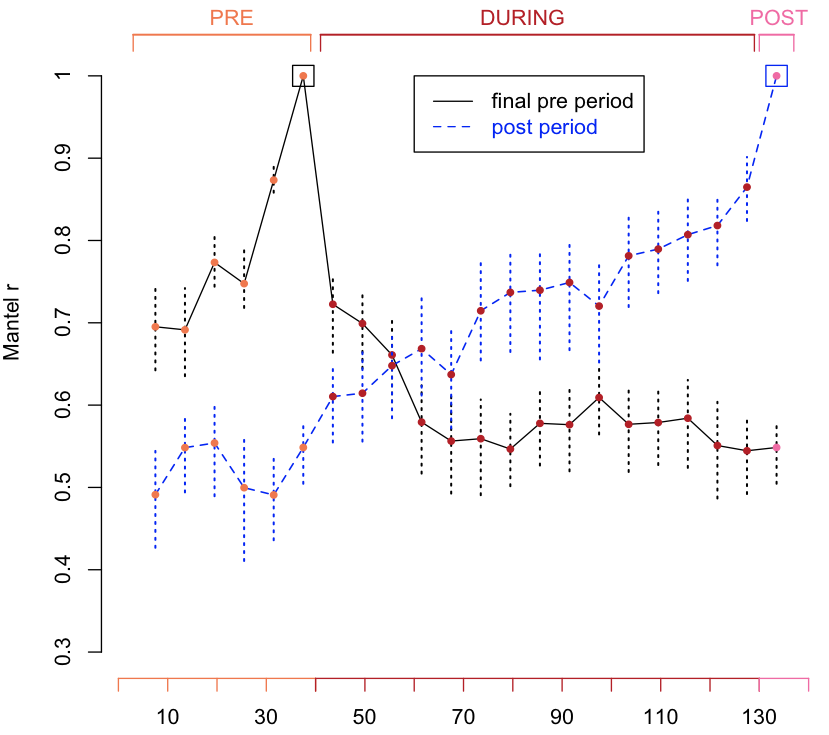


Figure SI 4. Mantel test results comparing each 6 day network to the final 6 day network of the pre-experimental period (solid black line) and to the 6 day post experimental network (dashed blue line), but only considering the 54 individuals that were observed in all 6-day periods (in comparison to Figure 3, which includes all individuals). Point colour denotes experimental period. Boxes show point of comparison. Vertical dotted lines indicate 95% range of Mantel test statistic.


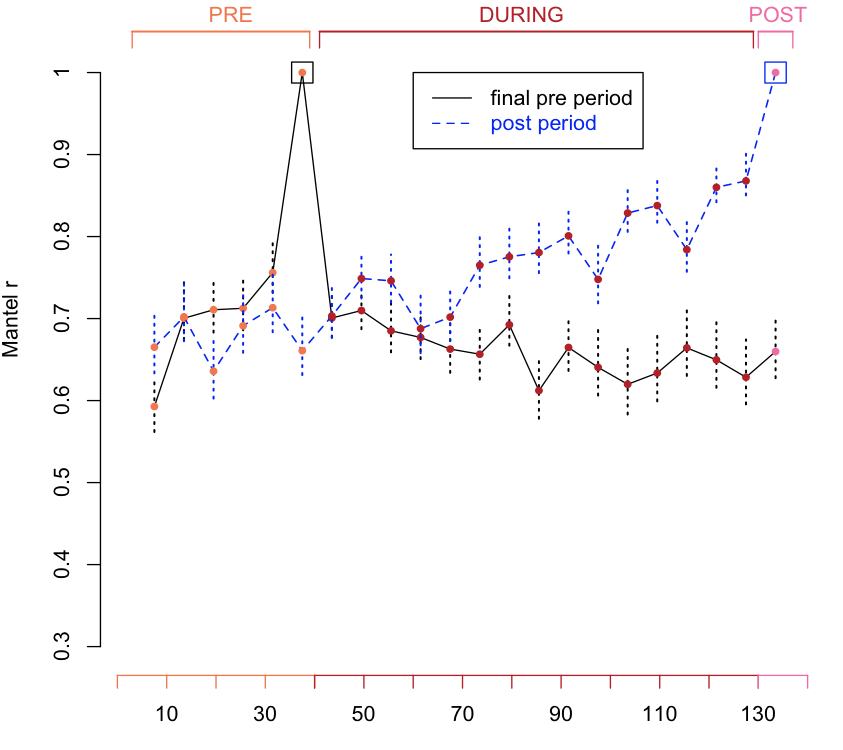


Figure SI 5. Mantel test results comparing each 6 day spatial range overlap matrix to the final 6 day spatial range overlaps of the pre-experimental period (solid black line) and to the 6 day post experimental spatial range overlaps (dashed blue line). Point colour denotes experimental period. Boxes show point of comparison. Vertical dotted lines indicate 95% range of Mantel test statistic.
